# Editorial: Exploring sources and mitigation strategies for science anxiety in educational contexts

**DOI:** 10.3389/fpsyg.2025.1674790

**Published:** 2025-10-01

**Authors:** Emma C. Burns, Lawrence Grabau

**Affiliations:** ^1^Macquarie University, Sydney, NSW, Australia; ^2^University of Kentucky, Lexington, KY, United States

**Keywords:** science anxiety, mathematics anxiety, STEM anxiety, mitigation strategies, teacher behaviors, student characteristics

Science anxiety is a growing concern that may impede student learning and literacy in science fields, as well as in STEM subjects more broadly, across various educational levels and school systems. Science anxiety refers to heightened fear or negative affect and arousal during science class or while engaging in science tasks (Mallow et al., 2010). This anxiety is often linked to both science tasks (learning) and science evaluation (testing). Recent studies, such as those utilizing the abbreviated science anxiety scale, have identified two distinct aspects of science anxiety related to learning and testing, both of which negatively impact science achievement. Despite these findings, there remains a significant gap in understanding the underlying variables—such as student, school, and cultural factors—that contribute to science anxiety. Addressing these gaps could lead to the development of practical, contextually tailored interventions aimed at mitigating science anxiety. Additionally, the interplay between science anxiety and other forms of anxiety, such as generalized anxiety, math anxiety, and social anxiety, particularly in the context of stressful life events like the COVID-19 pandemic, warrants further investigation.

This Research Topic, thus, aimed to explore the sources, impacts, co-variates, and mitigation strategies for STEM-related anxieties. The primary objectives included identifying the underlying factors contributing to science anxiety, understanding how these factors differ across various educational contexts and different pedagogies, and developing effective interventions. A key take-away across the studies included in this Research Topic is the potential impact of innovative pedagogies to help reduce anxiety without reducing academic standards. For example, there appears to be value in adopting strategies that can mitigate perceptions of “high stakes”—be it academic or social—which can allay anxiety in ways that enable better learning. Further research into the teaching strategies and how best to prepare teachers to consider these practices is an exciting avenue of future study.

The four articles included in this Research Topic are described briefly below, providing a sense of what the authors were investigating in their research work. All four articles relate STEM anxieties to STEM-related outcomes; however, each of those articles is tied to a particular context, ranging from calculus assessment to spatial learning to active learning environments to international comparisons.

Zakariya et al., concerned about test anxiety faced by Norwegian university students on calculus examinations, evaluated student perceptions of a newly implemented assessment strategy. Rather than a single, end-of-course, high-stakes examination, students took four online tests, each allowing multiple attempts, along with an individual project. Student perceptions were generally favorable; they reported more consistent preparation, lower test anxiety, and positive collaborative learning experiences. The authors suggest that this low-stakes approach to calculus assessment should be more widely considered to enhance students' learning environment.

Rocha et al. considered whether teachers of primary school students may choose spatial skill learning experiences based on their own spatial skills and spatial affect. Thus, their assessment was of K-6 teachers in the U.S., revealing that teachers with more highly developed spatial skills also exhibited spatial habits of mind and preferred more developed spatial strategies to help their students learn science. In contrast, teachers' preferences for math pedagogies were more closely related to their math teaching experience. The authors argue for enhanced teacher education programs to better prepare pre-service teachers to build conducive learning environments related to students' spatial skills.

Li et al. investigated whether active learning environments could minimize anxiety levels of forestry students in a Chinese university and thus enhance student performance. While more active learning environments generally reduced anxiety, the authors reported that some pedagogies chosen to increase student engagement actually increased their anxiety. Calling on students individually (“cold calling”) appeared to increase students' anxiety levels, as did participation in group work; meanwhile, the use of “clicker” technology was not associated with anxiety levels. The authors suggested that cold calling was likely not a strong candidate for inclusion in more active learning environments. Surprisingly, they found that moderate student anxiety levels were associated with better test performance.

Grabau et al., following up on prior research of Taiwanese students, investigated the association between schoolwork-related anxiety and five proficiency levels of science literacy in two contrasting world regions: southeast Asia and northwest Europe. While both regions had above average science literacy, anxiety was well above the international mean in southeast Asia and well below that mean in northwest Europe. Non-linear relationships between schoolwork-related anxiety and science literacy were uncovered for several of the six southeast Asian nations/entities; meanwhile, two of the six northwest European nations showed a negative linear relationship between these measures. Possible strategies to mitigate non-adaptive schoolwork-related anxiety among intermediate performance levels are suggested.
